# Association of the G8 score with urinary continence recovery after robot-assisted radical prostatectomy

**DOI:** 10.1007/s00345-026-06221-7

**Published:** 2026-01-30

**Authors:** Sang Won So, Seung-hwan Jeong, Hyeong Dong Yuk, Ja Hyeon Ku, Cheol Kwak, Jang Hee Han, Chang Wook Jeong

**Affiliations:** Department of Urology, Seoul National University Hospital, Seoul National University College of Medicine, 101, Daehak-ro, Jongno-gu, Seoul, 03080 Republic of Korea

**Keywords:** G8 questionnaire, RARP, Continence, Frailty, Nerve-sparing

## Abstract

**Purpose:**

To examine the association between the preoperative G8 score and urinary continence recovery after robot-assisted radical prostatectomy (RARP).

**Methods:**

This study included 1794 patients enrolled in SUPER-PC-RP prospective cohort who underwent RARP and completed G8 questionnaire preoperatively. Patients were classified into high (G8 score ≤ 14) and low (G8 score > 14) frailty groups. Continence recovery, defined as the use of less than one pad per day, was assessed three months and one year postoperatively. Factors affecting continence recovery were identified using multivariable logistic analysis. Kaplan-Meier analysis evaluated continence recovery over two years according to frailty and nerve-sparing status.

**Results:**

Overall, 649 and 1145 patients were assigned to high- and low-frailty groups, respectively. At three months, continence group was significantly younger and had a lower prevalence of diabetes, lower International Society of Urological Pathology grade, lower pathological T stage, higher nerve-sparing rate, and lower frailty than incontinence group. At one year, patients who recovered continence were younger and had a higher nerve-sparing rate and lower frailty. Multivariable analysis showed age (3-month odds ratio (OR) 0.973, 1-year OR 0.947), nerve-sparing (3-month OR 2.39, 1-year OR 1.77), and low frailty (3-month OR 1.56, 1-year OR 1.75) as significant factors affecting continence recovery (all *P* < 0.05). In cumulative Kaplan-Meier analysis, low-frailty group showed better continence recovery than high-frailty group, with a greater difference observed in non-nerve-sparing group (*P* < 0.001).

**Conclusion:**

Frailty assessment using G8 questionnaire is associated with urinary continence recovery after RARP, which provides an advantage for preoperative patient counseling and surgical planning.

**Supplementary Information:**

The online version contains supplementary material available at 10.1007/s00345-026-06221-7.

## Introduction

Most patients with prostate cancer experience postoperative urinary incontinence after radical prostatectomy because of pelvic floor muscle weakening, urethral sphincter injury, continence-related nerve damage [1–3]. Despite advances in robot‑assisted radical prostatectomy (RARP), urinary incontinence remains a major determinant of postoperative quality of life and an important drawback of surgery compared with radiation therapy or androgen deprivation therapy [4].

Previous studies for predicting continence recovery after RARP have focused on multiple factors, including patient factors (age, body mass index(BMI), and membranous urethral length) [5–7], disease factors (prostate-specific antigen, Gleason grade, and pathologic stage), and surgical factors (nerve-sparing technique, Retzius-sparing approach, and surgeon experience) [8–11]. Nevertheless, the impact of preoperative frailty has been less well explored.

The G8 questionnaire provides a simple way to quantify frailty and overall health status [12–15]. It is an eight‑item screening tool that reflects food intake, mobility, medication, and BMI, recommended for prostate cancer patients in guidelines [16, 17]. Previous study in our center examined the relationship between the G8 questionnaire and complications after uro-oncologic surgery [18]. However, no large-scale study has reported a correlation between preoperative G8 questionnaire scores and continence recovery after RARP.

This study aimed to determine whether the G8 score is related to continence recovery after RARP and to compare its usefulness across postoperative periods.

## Materials and methods

### Study population

This study involved patients registered in Seoul National University Prospectively Enrolled Registry for Prostate Cancer-Radical Prostatectomy (SUPER-PC-RP), with the approval of Seoul National University Hospital Institutional Review Board (SNUH IRB) (No. 1506-121-682) [19]. 3704 patients, enrolled in this prospective cohort, underwent radical prostatectomy for prostate cancer at SNUH between March 2016 and May 2024.

Among the patients in this cohort, 3290 patients who underwent RARP were initially extracted, and 1794 patients who completed G8 questionnaire preoperatively were included in this study (Supplementary Table 1). This observational study was approved by SNUH IRB (No. 2412-095-1596). All patients underwent conventional six-port RARP by seven experienced surgeons with van Velthoven urethrovesical anastomosis and both anterior and posterior reconstructions [9]. Patients with urinary incontinence history before surgery were excluded.

## Study design

Baseline characteristics including age, BMI, and comorbidities such as hypertension and diabetes, were examined. Disease factors were investigated, including preoperative PSA, International Society of Urological Pathology (ISUP) Grade Group, and pathologic T stage. For RARP, the presence of positive surgical margin and the use of nerve-sparing technique were investigated.

The G8 questionnaire was filled out by patients in the outpatient clinic before surgery. Based on previous studies and recommendations of International Society of Geriatric Oncology (SIOG), patients were divided into high-frailty group (≤ 14 points) and low-frailty group (˃14 points) [13, 16]. The treatment plan was not changed based on the preoperative questionnaire.

After RARP, postoperative incontinence status was checked in outpatient clinic at least once every three months. Recovery of urinary continence was defined as the use of less than one pad per day [20]. The time criterion for early continence was defined as three months post-surgery [4]. At three months and one year after surgery, patients were divided into continence and incontinence groups, respectively. The ratios of high- and low-frailty groups were compared for each parameter, excluding patients with follow-up loss. During two-year postoperative follow-up period, the proportion of patients recovering from urinary incontinence was analyzed according to each frailty group and nerve-sparing status.

### Statistical analysis

Data analyses were performed using XLSTAT Life Science software. Age and BMI were analyzed using two-tailed t-test. Preoperative PSA levels were analyzed using Mann-Whitney U test. Other parameters, including comorbidities, disease factors, and surgical factors, were analyzed using chi-square test. Multivariable logistic regression analysis was performed to analyze factors related to continence recovery at three months and one year post-surgery, respectively. Kaplan-Meier analysis evaluated the postoperative time to continence recovery comparing each frailty group and nerve-sparing status. Statistical significance was set at *P*-value < 0.05.

## Results

Among 1794 patients, 1652 patients were examined for continence recovery three months post-surgery, and 1229 patients were investigated for continence recovery one year post-surgery (Supplementary Fig. 1). 1049 patients (64%) had early continence recovery at postoperative 3-month, who were younger (73.6 vs. 74.3; *P* = 0.002), had a lower rate of diabetes mellitus (20% vs. 25%; *P* = 0.025), and had lower preoperative PSA (7.4 ng/mL vs. 8.2 ng/mL; *P* = 0.001) than those who remained incontinent (Table 1). Conversely, patients without early continence recovery had a higher rate of ISUP Grade Group 4‒5 (12% vs. 8%; *P* = 0.008) and pathologic T3‒T4 (44% vs. 38%; *P* = 0.016). The rate of nerve-sparing performed was higher in early continence group (84% vs. 66%; *P* < 0.001). The proportion of patients in low-frailty group was higher in early continence group (68% vs. 58%; *P* < 0.001) and lower in incontinence group (32% vs. 42%; *P* < 0.001).

**Table 1:** Patient characteristics depending on 3 months (early continence) and 1 year continence

	3 months (early continence) (*n* = 1652)			1 year (*n* = 1229)		
	Continence (*n* = 1049)	Incontinence (*n* = 603)	*P*-value	Continence (*n* = 1125)	Incontinence (*n* = 104)	*P*-value
Age (yrs) (mean ± SD)	73.6 ± 4.5	74.3 ± 4.6	0.002	74.1 ± 4.6	75.5 ± 4.3	0.002
BMI (kg/m^2^) (mean ± SD)	24.7 ± 2.8	24.5 ± 2.9	0.273	24.7 ± 2.7	24.3 ± 2.7	0.198
HTN (n,%)	557 (53)	333 (55)	0.404	596 (53)	52 (50)	0.561
DM (n,%)	213 (20)	151 (25)	0.025	235 (21)	26 (25)	0.327
PSA (ng/dl) (median [IQR])	7.4 [5.1, 12.5]	8.2 [5.8, 14.7]	0.001	7.5 [5.2, 12.9]	8.7 [6.4, 12.9]	0.05
ISUP Grade Group (n, %)			0.008			0.438
Grade Group 1–3	891 (92)	445 (88)		943 (90)	83 (87)	
Grade Group 4–5	79 (8)	63 (12)		106 (10)	12 (13)	
Pathologic T stage (n, %)			0.016			0.714
T1-2	602 (62)	283 (56)		619 (59)	58 (61)	
T3-4	366 (38)	225 (44)		428 (41)	37 (39)	
Positive surgical margin (n, %)	319 (33)	149 (29)	0.163	332 (31)	23 (24)	0.133
Nerve sparing (n, %)			< 0.001			0.003
Non-nerve sparing	162 (16)	202 (34)		219 (19)	33 (32)	
Nerve sparing	886 (84)	401 (66)		905 (81)	71 (68)	
G8 score (n, %)			< 0.001			0.002
High frailty group (≤ 14)	337 (32)	251 (42)		364 (32)	49 (47)	
Low frailty group (> 14)	712 (68)	352 (58)		761 (68)	55 (53)	

BMI = body mass index, DM = diabetes mellitus, HTN = hypertension, PSA = Prostate-specific antigen, ISUP = The International Society of Urological Pathology

At postoperative 1-year, 1125 patients (92%) achieved continence recovery (Table 1). Patients without continence recovery within one year were significantly older (75.5 vs. 74.1; *P* = 0.002) and underwent nerve-sparing less frequently (68% vs. 81%, *P* = 0.003) than those who recovered continence. Furthermore, low-frailty patients were more observed at continence group than incontinence group (68% vs. 53%; *P* = 0.002).

Considering that several parameters affected continence recovery after RARP, a multivariable logistic regression analysis among these parameters was performed for each period (Table 2). The association of young age with faster continence recovery was retained, regardless of follow-up period (3 months: odds ratio (OR) 0.973, 95% CI 0.950‒0.997, *P* = 0.029 vs. 1 year: OR 0.947, CI 0.906‒0.989, *P* = 0.014). The nerve-sparing method also significantly affected continence recovery at three months (OR 2.39, CI 1.82‒3.14, *P* < 0.001) and one year (OR 1.77, CI 1.13‒2.76, *P* = 0.012) postoperatively. In particular, low frailty tended to improve continence recovery regardless of the postoperative period (3 months: OR 1.56, CI 1.24‒1.96, *P* < 0.001 vs. 1 year: OR 1.75, CI 1.17‒2.65, *P* = 0.007).

**Table 2:** Multivariable logistic regression analysis of 3 months & 1 year continence after Robot-assisted radical prostatectomy

	3 months (early continence)			1 year		
	OR	CI	*P*-value	OR	CI	*P*-value
Age	0.973	0.950–0.997	0.029	0.947	0.906–0.989	0.014
DM	0.779	0.598–1.014	0.064			
Pathologic T3-T4	0.881	0.700-1.109	0.280			
ISUP Grade Group 4–5	0.790	0.544–1.147	0.215			
Nerve sparing method	2.39	1.82–3.14	< 0.001	1.77	1.13–2.76	0.012
Low frailty	1.56	1.24–1.96	< 0.001	1.75	1.17–2.65	0.007

DM = diabetes mellitus, ISUP = The International Society of Urological Pathology

Additional multivariable logistic regression analysis was performed considering the G8 score as a continuous variable (Supplementary Table 2). The same variables listed in Table 2 were used in additional analysis. The G8 score was significantly associated with continence recovery at each period (3-month OR 1.17, CI 1.09–1.25, *P* < 0.001; 1-year OR 1.23, CI 1.09–1.38, *P* < 0.001). The G8 score showed significant results even when it was considered as a continuous variable as well as a dichotomous variable.

**Fig. 1:**
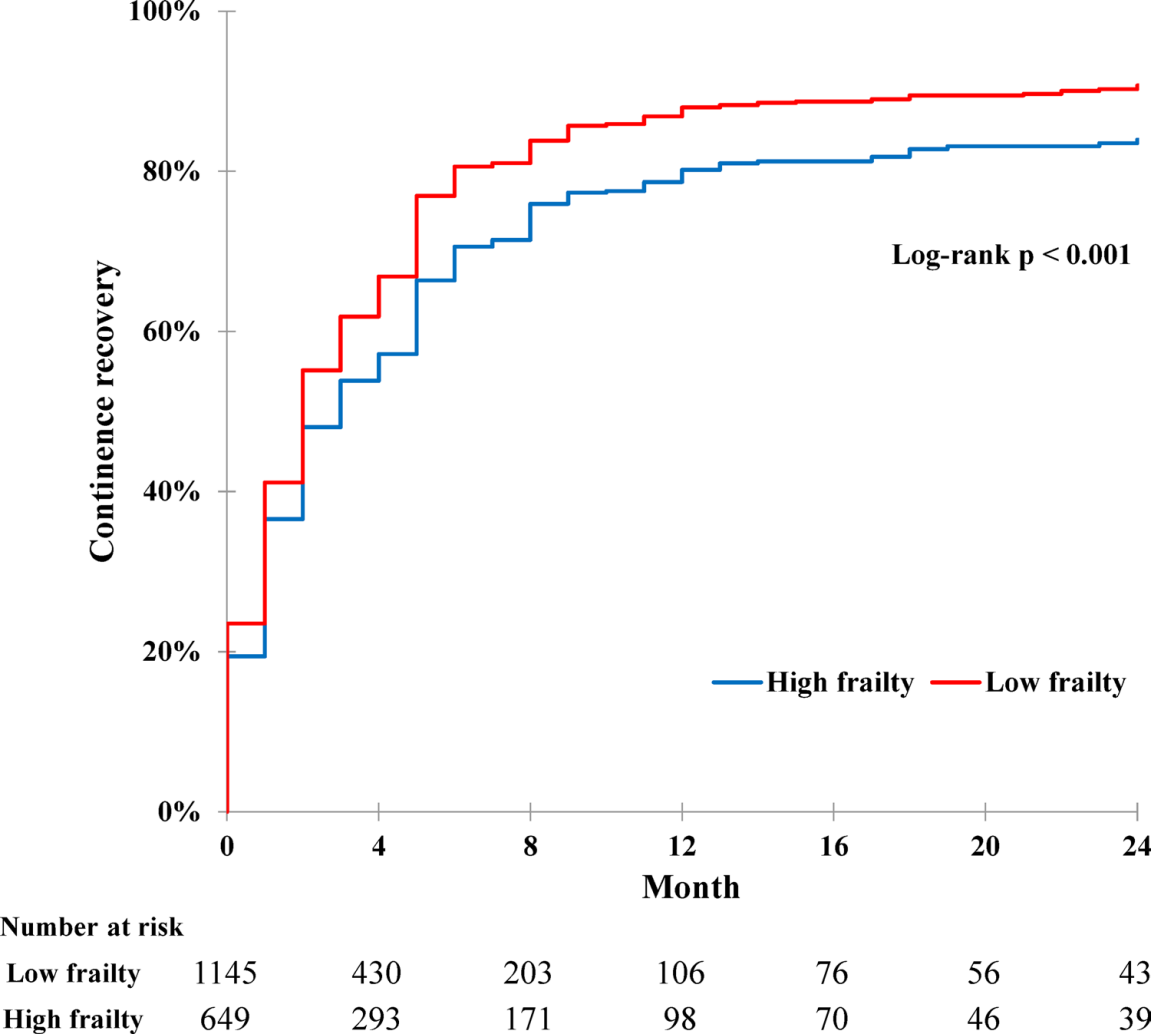
Cumulative Kaplan-Meier analysis on postoperative continence recovery rate with comparing high frailty group (blue line) and low frailty group (red line) after Robot-assisted radical prostatectomy

In Kaplan-Meier analysis with the results expressed as the cumulative incidence (Fig. 1), patients in low-frailty group showed faster continence recovery than high-frailty group (*P* < 0.001). To analyze continence recovery time according to nerve-sparing, additional Kaplan-Meier analysis was performed on the subgroups with nerve-sparing and without nerve-sparing (non-nerve-sparing) (Supplementary Fig. 2A, B). Continence recovery in low-frailty group was faster than high-frailty group in both nerve-sparing (*P* < 0.001) and non-nerve-sparing (*P* = 0.003) groups. Comparing non-nerve-sparing RARP at low frailty with nerve-sparing RARP at high frailty (Supplementary Fig. 2C), the latter group experienced faster continence recovery than the former group until one year postoperatively (*P* = 0.026). However, this tendency was reversed one year after surgery.

## Discussion

For patients with prostate cancer, radical prostatectomy should carefully balance oncological control with postoperative quality of life, particularly urinary incontinence [21]. In this cohort, preoperative frailty assessed using the G8 questionnaire was independently associated with continence recovery after RARP; patients with higher frailty exhibited significantly lower continence recovery rates across postoperative follow-up period. Our finding is consistent with Stankovic et al., who reported that frail patients, classified according to the Clinical Frailty Scale, had poorer early continence recovery 30 days after radical prostatectomy regardless of surgical approach [22]. Our study extends these results by demonstrating the sustained impact of frailty on continence recovery over two years in a larger prospective RARP cohort, using a more detailed and user-friendly questionnaire that is recommended in guidelines and also widely applied in prostate and other cancers [14, 16, 18].

In our study, we adopted “less than one pad per day” definition, which corresponds to social continence commonly applied in prior literatures distinctly from strict “0-pad” definition [10, 23]. To compare continence recovery according to the two definitions, additional Kaplan-Meier analysis was performed using “0-pad” definition as the criterion and frailty as the subgroup (Supplementary Fig. 3). Our data showed that, within the same cohort, “0-pad” definition showed slower recovery of continence than our definition (*P* < 0.001). Regardless of the definition, low frailty showed significantly faster continence recovery than high frailty (*P* < 0.001).

There was concern that the results of minor experienced surgeons, who performed RARP less than 400 cases, would affect the analysis of functional outcomes despite the small number (10.6%) included. However, compared to the highly experienced group, minor experienced group showed homogenous G8 score distribution despite their limited experience (minor experienced 14.7 ± 1.8 vs. highly experienced 14.7 ± 1.7), and was not inferior in terms of nerve sparing technique (minor experienced 84% vs. highly experienced 78%). The functional outcome was also noninferior in minor experienced group (1-year continence recovery rate; minor experienced 94% vs. highly experienced 91%).

Our study suggests that frailty has a comparable, and in some contexts greater, association with continence recovery than other perioperative factors. It is well established that nerve-sparing improves continence recovery [24, 25], and in our multivariable analysis nerve-sparing was associated with better early postoperative continence although this effect weakened by one year (3-months OR 2.39, 1-year OR 1.77; Table 2). In contrast, the association of low frailty with continence recovery became slightly stronger over time (3-months OR 1.56, 1-year OR 1.75).

Subgroup analyses further demonstrated that the magnitude and persistence of the nerve-sparing effect differed according to frailty status (Supplementary Fig. 4). At 3-months postoperatively, patients in high-frailty group derived greater relative benefit from nerve-sparing (OR 3.27, CI 2.20‒4.85; *P* < 0.001) than those in low-frailty group (OR 2.46, CI 1.82‒3.33; *P* < 0.001). Conversely, at 1-year, the effect of nerve-sparing on continence recovery was attenuated in the high-frailty group (OR 1.49, CI 0.76‒2.91; *P* = 0.242), whereas it remained significant in low-frailty group (OR 2.30, CI 1.28‒4.12; *P* = 0.005). This pattern was consistent with the cumulative Kaplan–Meier analysis comparing nerve-sparing in high-frailty patients with non–nerve-sparing in low-frailty patients (Supplementary Fig. 2C), which showed a reversed trend in continence recovery at one year.

These findings align with the meta-analysis by Reeves et al., which indicated that nerve-sparing markedly improves continence recovery within six months after radical prostatectomy, with no significant difference beyond 12 months compared with non–nerve-sparing [23]. Early benefit from nerve-sparing reflects preservation of somatic innervation to the external urethral sphincter along with the pudendal nerve, whereas later recovery appears to depend more on compensatory mechanisms such as pelvic floor muscles; this compensation seems less effective in patients with high frailty, which explain the greater attenuation of nerve-sparing effect in this group. Taken together, our results indicate that although nerve-sparing is important for early functional recovery, preoperative frailty status is a key determinant of sustained urinary continence after RARP and should be carefully considered in patient counselling.

A noteworthy aspect of our study is its clinical utility for high-risk or locally advanced prostate cancer. In cases of ISUP grades 4–5 or tumors extending beyond prostate capsule, oncologic outcomes should be considered rather than nerve-sparing. This involves sacrificing the surrounding neurovascular bundle, increasing the risk of postoperative incontinence and erectile dysfunction. In such situations, frailty evaluation with G8 score is a valuable reference for surgical strategies and preoperative patient consultations. Indeed, the rate of persistent urinary incontinence was high among patients with high-grade and high-T stage disease at three months postoperatively; however, most patients with incontinence at three months (81% with high-grade and 84% with high-T stage) recovered within one year (Table 1). In addition, low-frailty patients showed improved continence recovery rates even in the absence of nerve-sparing (Supplementary Fig. 2). Therefore, our results confirm that continence recovery in patients with low frailty will not be considerably hindered even if aggressive resection is performed, considering oncological outcomes as the top priority.

Our study had several limitations. First, this study did not include an analysis of erectile function, which is considered in conjunction with urinary incontinence when nerve-sparing is performed during RARP. Second, pelvic floor muscle training protocol which accelerates continence recovery after RARP has not yet been formalized in our center. It is possible that patients were not able to perform pelvic floor muscle training equally due to the absence of a structural protocol. Third, there was a selection bias, because the study included patients who had been followed up for less than one year after surgery; 74% of the patients followed up at 3 months were included in the 1-year follow-up, and consecutive analysis for some patients was unavailable. Finally, several functional variables known to affect postoperative continence recovery—such as preoperative lower urinary tract symptoms, prostate size, membranous urethral length, and pelvic floor muscle status—were not available in our cohort and therefore were not analyzed. Future studies integrating both frailty status and detailed functional parameters may further refine individualized prediction models for continence recovery.

Nevertheless, our study provides meaningful evidence in that frailty, assessed by a simple quantitative screening tool, is associated with postoperative continence recovery. This association aids in treatment selection and preoperative counseling, and also supports the SIOG recommendation that older patients with prostate cancer should be managed based on health status rather than age alone [12]. In cases of low frailty with advanced prostate cancer, this assessment provides a basis for integrating oncological considerations, whereas in low-stage tumors with high frailty, it provides a valuable reference for selecting appropriate treatment strategies. In this respect, our results contribute to establishing individualized surgical strategies and patient counseling tailored to the degree of frailty.

## Conclusion

In conclusion, preoperative G8 questionnaire score is associated with postoperative urinary continence recovery in patients who underwent RARP.

## Supplementary Information

Below is the link to the electronic supplementary material.

Supplementary Material 1

## Data Availability

No datasets were generated or analysed during the current study.
