# Supplementary material for: Association of the G8 score with urinary continence recovery after robot-assisted radical prostatectomy
Source: World J Urol. 2026 Jan 30;44(1):140. doi: 10.1007/s00345-026-06221-7 (PMC12858616; doi:10.1007/s00345-026-06221-7)
Supplement: Supplementary file 1 — Supplementary Material 1 [file 345_2026_6221_MOESM1_ESM.docx]

**Supplementary Table 1.** Baseline patient characteristics (n=1794)

| **Characteristics** | **All (n=1794)** |
| --- | --- |
| Age (yrs)  BMI (kg/m^2^)  HTN (n,%)  DM (n,%) | 73.8±4.6  24.6±2.8  968 (54.0)  394 (22.0) |
| PSA (ng/dl) | 7.7 [5.4, 13.2] |
| ISUP Grade Group (n, %) |  |
| Grade Group 1-3 | 1442 (90.5) |
| Grade Group 4-5 | 151 (9.5) |
| Pathologic T stage (n, %) |  |
| T1-2 | 955 (60.0) |
| T3-4 | 636 (40.0) |
| Positive surgical margin (n, %) | 511 (28.5) |
| Nerve sparing (n, %) |  |
| Non-nerve sparing | 385 (21.5) |
| Nerve sparing | 1408 (78.5) |
| G8 score (n, %) |  |
| High frailty group (≤14) | 649 (36.2) |
| Low frailty group (>14) | 1145 (63.8) |

BMI=body mass index, DM=diabetes mellitus, HTN=hypertension, PSA=Prostate-specific antigen, ISUP=The International Society of Urological Pathology

**Supplementary Table 2.** Multivariable logistic regression analysis of 3 months & 1 year continence after Robot-assisted radical prostatectomy, with considering G8 questionnaire score as continuous variable.

|  | **3 months (Early continence)** | | |  | **1 year** |  |
| --- | --- | --- | --- | --- | --- | --- |
|  | **OR** | **CI** | ***P*-value** | **OR** | **CI** | ***P*-value** |
| Age  DM  Pathologic T3-T4  ISUP Grade Group 4-5  Nerve sparing method  G8 score | 0.975  0.801  0.882  0.783  2.32  1.17 | 0.951-0.999  0.614-1.044  0.700-1.110  0.539-1.137  1.77-3.05  1.09-1.25 | 0.041  0.101  0.284  0.199  <0.001  <0.001 | 0.956  1.68  1.23 | 0.914-1.001  1.04-2.72  1.09-1.38 | 0.055  0.034  <0.001 |

DM=diabetes mellitus, ISUP=The International Society of Urological Pathology

**Supplementary Figure 1.** Patient flow diagram **Supplementary Figure 2.** Cumulative Kaplan-Meier analysis on postoperative continence recovery rate with comparing high frailty group (blue line) and low frailty group (red line) after Robot-assisted radical prostatectomy (RARP); (A) Nerve Sparing RARP, (B) Non-Nerve Sparing RARP and (C) Nerve Sparing at High Frailty vs Non-Nerve Sparing at Low Frailty

**Log-rank p < 0.001**

| **Number at risk** | | |  | |  | |  | |  | |  | |  |
| --- | --- | --- | --- | --- | --- | --- | --- | --- | --- | --- | --- | --- | --- |
| **0-pad definition** | |  | |  | |  | |  | |  | |  | |
| Low frailty | 1145 | 785 | | 484 | | 274 | | 204 | | 139 | | 111 | |
| High frailty | 649 | 468 | | 317 | | 206 | | 157 | | 112 | | 95 | |
| **<1-pad definition** | |  | |  | |  | |  | |  | |  | |
| Low frailty | 1145 | 430 | | 203 | | 106 | | 76 | | 56 | | 43 | |
| High frailty | 649 | 293 | | 171 | | 98 | | 70 | | 46 | | 39 | |

**Supplementary Figure 3.** Cumulative Kaplan-Meier analysis on postoperative continence recovery rate according to each frailty group, with comparing “less than one pad per day(<1-pad)” definition and “0-pad” definition after Robot-assisted radical prostatectomy. Solid lines refer to “less than one pad per day(<1-pad)” definition while dotted lines refer to “0-pad” definition, for each frailty group; high frailty group (blue line) and low frailty group (red line).

**Supplementary Figure 4.** Effect of Nerve sparing technique on Continence recovery by Postoperative period and Frailty group
